# Association between cortisol levels and performance in clinical simulation: a systematic review

**DOI:** 10.1590/1980-220X-REEUSP-2023-0279en

**Published:** 2024-07-26

**Authors:** Jackson Gois Teixeira, Lucas Tomaz Benigno Lima, Elaine Carvalho Cunha, Flavia Oliveira de Almeida Marques da Cruz, Karen Karoline Gouveia Carneiro, Laiane Medeiros Ribeiro, Guilherme da Costa Brasil

**Affiliations:** 1 Centro Universitário do Distrito Federal, Departamento de Enfermagem, Brasília, DF, Brazil.; 2 Universidade de Brasília, Programa de Pós-Graduação em Enfermagem, Brasília, DF, Brazil.; 3 Centro Universitário do Planalto Central Apparecido dos Santos, Departamento de Enfermagem, Brasília, DF, Brazil.; 4 Universidade de Brasília, Departamento de Enfermagem, Brasília, DF, Brazil.

**Keywords:** Employee Performance Appraisal, Stress, Psychological, Hydrocortisone, Simulation Training, Systematic Review, Evaluación del Rendimiento de Empleados, Estrés Psicológico, Hidrocortisona, Entrenamiento Simulado, Revisión Sistemática

## Abstract

**Objective:**

To identify how stress measured by salivary cortisol during clinical simulation-based education, or simulation and another teaching method, impacts performance.

**Method:**

Systematic review of the association between cortisol and performance in simulations. The following databases were used: PubMed, LIVIVO, Scopus, EMBASE, Latin American and Caribbean Health Sciences Literature (LILACS) and Web of Science. Additional searches of gray literature were carried out on Google Scholar and Proquest. The searches took place on March 20, 2023. The risk of bias of randomized clinical trials was assessed using the Cochrane Collaboration Risk of Bias Tool (RoB 2). Inclusion criteria were: simulation studies with salivary cortisol collection and performance evaluation, published in any period in Portuguese, English and Spanish.

**Results:**

11 studies were included which measured stress using salivary cortisol and were analyzed using descriptive synthesis and qualitative analysis.

**Conclusion:**

Some studies have shown a relationship between stress and performance, which may be beneficial or harmful to the participant. However, other studies did not show this correlation, which may not have been due to methodological issues.

## RESUMEN

**Objetivo:**

Identificar cómo el estrés medido por el cortisol salival durante la enseñanza basada en la simulación clínica, o la simulación y otro método de enseñanza, influye en el rendimiento.

**Método:**

Revisión sistemática de la asociación entre cortisol y rendimiento en simulaciones. Se utilizaron las siguientes bases de datos: PubMed, LIVIVO, Scopus, EMBASE, Literatura Latinoamericana y del Caribe en Ciencias de la Salud (LILACS) y Web of Science. Se realizaron búsquedas adicionales de literatura gris en Google Scholar y Proquest. Las búsquedas se realizaron el 20 de marzo de 2023. El riesgo de sesgo de los ensayos clínicos aleatorios se evaluó mediante la Herramienta de Riesgo de Sesgo (RoB 2) de la Colaboración Cochrane. Los criterios de inclusión fueron: estudios de simulación con recogida de cortisol salival y evaluación del rendimiento, publicados en cualquier periodo en portugués, inglés y español.

**Resultados:**

Se incluyeron 11 estudios que midieron el estrés mediante cortisol salival y se analizaron mediante síntesis descriptiva y análisis cualitativo.

**Conclusión:**

Algunos estudios mostraron una relación entre el estrés y el rendimiento, que puede ser beneficiosa o perjudicial para el participante. Sin embargo, otros estudios no mostraron esta correlación, lo que puede no haberse debido a cuestiones metodológicas.

## INTRODUCTION

Simulation in health has become a training method explored in teaching laboratories and simulation centers, with the aim of developing technical and non-technical skills, bringing benefits to the learning process and contributing to professional training and improvement^(1,2)^.

Realistic simulation has been described as a stressful experience^(3)^. Stress is associated with negative cognitive impacts, such as decreased concentration, memory degradation, increased errors and delayed response to stimuli^(4)^. However, and up to a specific point, stress may improve concentration on the task, focus on communication and contribute to problem-solving^(5)^. Furthermore, circumstances perceived as threatening tend to trigger negative emotions, while evaluations of challenge are correlated with more positive emotional responses^(6)^.

Furthermore, in a randomized clinical trial that investigated adding emotional stressors in a simulation, participants were able to recall the events of the scenarios that failed, indicating that emotional stress can improve the ability to recall these memories^(7)^. For that reason, moderate levels of stress are essential for effectiveness in active student learning^(8)^.

However, the causal meaning of the relationship remains undetermined: is high performance associated with a lower experience of stress, or is the constant presence of stress in an individual associated with a lower tendency to make mistakes^(9)^. It is also unclear whether additional stressors can have any detrimental effect on performance, since performance limitations can have an immediate effect on the quality of care provided to patients^(10)^.

The literature defines stress as a state of divergence between perceived demands, the individual’s reactions and the ability to adapt to stressors^(3)^ and is closely linked to emotions, involving emotional and physiological responses to a stressor^(11)^. Furthermore, stressful conditions trigger the activation of the endocrine, nervous and immune systems, a phenomenon widely recognized as a stress response^(12)^.

The human body allows to find biochemical markers of stress. Cortisol is a stress hormone produced in the adrenal cortex, and its concentration in saliva is strongly correlated with its concentration in blood plasma^(3)^. Salivary cortisol levels have been used extensively as an objective measure of stress in simulation, making it an ideal assay for research^(13)^. By acting as a biological marker, cortisol levels increase in response to stress and the numerous changes in the simulation environment^(8)^.

Initially, observational studies identified increases in participants’ cortisol levels^(14–17)^. However, a systematic review showed that the stress experienced in a simulation is still undefined^(18)^.

It is therefore important to synthesize the relationship between cortisol and the participant’s performance through a systematic review of intervention studies using group analysis, given that participants may have different physiological responses to different experiences and perceptions during simulations.

Against this backdrop, the aim of this study was to identify how stress measured by salivary cortisol during education based on clinical simulation or simulation and another teaching method, impacts on performance.

## METHOD

### Registration and Protocol

This is a systematic review studying the association between simulation and cortisol levels and performance, conducted in accordance with the recommendations of the Cochrane Collaboration^(19)^ and described in accordance with the Preferred Reporting Items for Systematic Reviews and Meta-Analyses - PRISMA^(20)^. The protocol was registered in the International Prospective Register of Systematic Reviews under the number CRD42022319886.

### Development of the Research Question

The question was guided by the PICO strategy, considering “P” (patient or problem) students or health professionals; “I” (intervention) realistic simulation; “C” (control) was not applied, and “O” (result or outcome) as the association between cortisol and performance. Thus, the guiding question was: What is the association between salivary cortisol levels and the performance of participants in simulation-based education?

### Eligibility Criteria

The systematic review included randomized clinical trials (RCTs) that assessed stress through salivary cortisol (SC) in the following contexts: (a) realistic simulations carried out with (medical., nursing) students; (b) simulations for training resident medical professionals; (c) simulations that included professionals from other health areas; (d) simulations within institutional laboratories – hospital setting; and (e) low, medium and high-fidelity simulation.

Studies were excluded due to the following criteria: (a) unavailability; (b) conference abstract; (c) virtual simulation; (d) not being an RCT; (e) not being developed in the context of realistic simulation; (f) assessing alpha amylase; (g) not assessing performance; (h) assessing anxiety.

### Databases and Search Strategy

The search was carried out in the following electronic databases: PubMed, LIVIVO, Scopus, EMBASE, Latin American and Caribbean Health Sciences Literature (LILACS), Web of Science (Social Sciences Citation Index). Additional searches of gray literature were carried out on Google Scholar and Proquest. The list of references of the selected studies was analyzed manually to identify potentially relevant ones that could have been missed in the electronic database searches. Duplicate references were removed using Rayyan^®^.

### Search Strategy

The search terms were adapted for use in the different electronic databases, combined with specific filters for controlled trials when available. Studies were selected in Portuguese, English and Spanish, and without a time cut-off to cover a broad spectrum of national and international publications. All the searches in the electronic databases were carried out on March 20, 2023, and the strategies are shown in Chart 1.

**Chart 1:** Search strategies in electronic databases – Brasília, DF, Brazil, 2024.

Database	Keywords
**PubMed**	#1 = (“Patient Simulation”[MeSH] OR “Simulation Training”[MeSH] OR “Simulation Training/methods”[MeSH]) OR “High Fidelity Simulation”[All Fields] OR “High Fidelity Simulation Training”[Mesh]) #2 = (“Stress, Psychological”[MeSH] OR “Stress, Physiological”[MeSH] OR “Stress Response”[All Fields] OR “Hydrocortisone”[MeSH] OR Cortisol[All Fields] OR “Salivary Cortisol”[All Fields] OR “Saliva/chemistry”[MeSH]) #3 = #1 AND #2
**LILACS**	(“Patient Simulation” OR “Simulation Training”) AND (“stress, psychological” OR “salivary cortisol”)
**ĹIVIVO**	(“Patient Simulation” OR “Simulation Training” OR “High Fidelity Simulation” OR “High Fidelity Simulation Training” OR “High-Fidelity Manikin” OR Simulation) AND (“Stress, Psychological” OR “Stress, Physiological” OR “Stress Response” OR “Hydrocortisone” OR Cortisol OR “Salivary Cortisol”)
**SCOPUS**	(ALL (“Patient Simulation” OR “Simulation Training” OR “High Fidelity Simulation” OR “High Fidelity Simulation Training” OR “High-Fidelity Manikin” OR simulation) AND TITLE-ABS-KEY (cortisol OR “Salivary Cortisol”) )
**Web of Science**	TS=((“Patient Simulation” OR “Simulation Training” OR “High Fidelity Simulation” OR “High Fidelity Simulation Training” OR “High-Fidelity Manikin” OR Simulation) AND TS=(“Stress, Psychological” OR “Stress, Physiological” OR “Stress Response” OR “Hydrocortisone” OR Cortisol OR “Salivary Cortisol”)
**EMBASE**	#1 = (“patient simulation”/exp OR “patient simulator”/exp OR “simulation training”/exp OR “high fidelity simulation training”/exp OR “high fidelity simulation”) #2 = (“psychological stress”/exp OR “Stress, Physiological” OR ‘mental stress’/exp “Stress Response” OR hydrocortisone/exp OR cortisol OR “salivary cortisol”) #3 = #1 AND #2
**Google Scholar**	(“Patient Simulation” OR “ Simulation Training” OR simulation) AND (“psychological stress” OR “cortisol”) Where my words occur: anywhere in the article 100 most relevant hits (10 pages)
**Proquest**	(“Patient Simulation” OR “Simulation Training” OR “Simulation Training/methods” OR “High Fidelity Simulation” OR “High Fidelity Simulation Training”) AND (“Stress, Psychological” OR “Stress, Physiological” OR “Stress Response” OR “Hydrocortisone” OR Cortisol OR “Salivary Cortisol” OR “Saliva/chemistry”)

### Selection Process

The studies were selected in two phases using the online application Rayyan^®^ (Qatar Computing Research Institute), a program that speeds up the initial screening of studies through a semi-automated process, which guarantees the reliability of the selection. In the first phase, two researchers independently examined the titles and abstracts of all the studies retrieved from the databases and identified those that met the inclusion criteria. In the second phase, the same researchers independently read the full text of all the selected studies and excluded those that did not meet the inclusion criteria. Any discrepancies at this stage would be resolved by discussion between the researchers and a specialist, who would also independently assess the study in full text.

### Data Collection Process

Two researchers independently extracted the data from the studies included in this systematic review, using a data collection instrument of their own creation. Any disagreements were resolved by discussion and mutual agreement. A third author was involved when necessary to make a final decision.

The variables collected included: characteristics of the participants (groups and sample); characteristics of the study (authors, country, year of publication, objective, design, randomization and inclusion and exclusion criteria); intervention (type of simulation, simulator, area of expertise); collection (cortisol measurement); and characteristics of the results (main results and main conclusions). If the necessary data was not complete, contact was made with the authors to obtain any relevant information. Based on this data, the results of this systematic review are presented descriptively in Table 1.

**Table 1:** General characteristics of the included studies according to year, author, country, groups, sample, objective, measurement of stress, cortisol, performance, area and conclusions – Brasília, DF, Brazil, 2024.

Year, author, country	Groups	N	Study characteristics / objective	Cortisol measurements	Cortisol	Performance	Area	Main conclusions
2017, Lizotte et al., Canada^(22)^.	IG: Simulation with death.	IG: 21	Evaluate the impact of simulations on trainees’ stress and performance; both during a “traditional” simulation (mannequin-survivors) and during a simulated death.	Salivary Cortisol.	T0: 0,10 µg/dL [IQR 0,07–0,14]. T1: 0,11 µg/dL [IQR 0,10–0,17]. T2: 0,17 µg/dL [IQR 0,13–0,28].	First scenario: 82 [IQR 78–88] = 0.85. Second scenario: 79 [IQR 77–86] = 0.87.	Medicine.	Neonatal simulation causes stress before and during the simulation without interfering with performance. Having a “dead” mannequin during a simulation does not increase objective stress or interfere with performance.
	CG: Simulation with survival	CG:21	T0: 0,10 µg/dL [IQR 0,06–0,15]. T1:0,15 µg/dL [IQR 0,09–0,22]. T2:0,23 µg/dL [IQR 0,14–0,47].		First scenario: 83 [IQR 74–89] = 0.85. Second scenario: 82 [IQR 72–88] = 0.87.			

2011, Keitel et al., Germany^(9)^.	IG: Simulated emergency situation.	34	To evaluate the psychological and endocrine responses to stress in realistic simulation and the relationship between performance and stress.	Salivary Cortisol.	–15 min: 0.25µg/dL (–0,25–0,75 IC). 0 min: 0.22 µg/dL (–0.27–0.72 IC). 15 min: 0.26 µg/dL (–0,24–0.76 IC). 30 min: 0.11 µg/dL (–0.39–0.61). 45 min: –0.15 µg/dL (–0.65–0.35 IC). 60 min: –0.25 µg/dL (–0.75–0.25 IC). 75 min: –0.10 µg/dL (–0.60–0.40 IC).	No significant correlation between increased salivary cortisol and performance (p = 0.811 and p = 0.631).	Medicine.	The positive relationship between endocrine stress response in a standard laboratory situation and performance in a simulated emergency situation indicates that high stress responsiveness can be a predictor of good performance.
	CG: Resting Condition.					correlated significantly with the increase in cortisol (p = 0.019).		

2016, Demaria et al., United States^(23)^.	IG: Simulation with death.	IG: 13	Describe the physiological and biochemical stress response between simulation with death and simulation with survival.	Salivary Cortisol.	0,193 µg/dL. The average increase in SC was 0.053 µg/dL [0.071 to 0.165].	83,3% [75–85,8] = (p = 0,18).	Medicine.	There was no negative response to a simulated patient death compared to simulated survival. Salivary cortisol increased compared to baseline levels, but there were no significant differences.
	CG: Simulation with survival.	CG: 13			0,159 µg/dL. 0,056 µg/dL [0,033–0,163] no statistical significant difference between groups	75% [64,1–84,2].		

2014, Piquette et al., Canadá^(24)^.	IG: Simulation with high stress scenario.	IG: 26	To explore the effects of modifiable external stressors on the simulated clinical performance of residents.	Salivary Cortisol.	Pre scenario (–15 min): 7.65 ± 5.19. Pre scenario (–5 min): 9.07 ± 6.39. Post scenario (0 min): 9.25 ± 7.17. Post scenario (10 min): 9.71 ± 7.00. Post scenario (20 min): 8.33 ± 5.08.	4.7 ± 0.9. 72% ± 11%.	Medicine.	There were significant physiological and psychological stress responses in the residents when they went through simulated resuscitation scenarios. Cortisol levels showed better performance in group A.
	CG: Simulation with low-stress scenario.	CG: 28			Pre scenario (–15 min): 7.11 ± 4.50. Pre scenario (–5 min): 8.14 ± 5.11. Post scenario (0 min): 8.64 ± 6.10. Post scenario (10 min): 9.20 ± 6.67. Post scenario (20 min): 7.70 ± 5.69.	4.9 ± 0.8. 70% ± 11%.		

2013, Meunier et al., Belgium^(25)^.	IG: trained residents.	IG: 50	To evaluate the effect of communication skills training on residents’ physiological arousal during the communication of bad news.	Salivary cortisol	Rest until end of preparation (before 32.4 ± 22.0 / after 44.9 ± 28.0). End of preparation to end of simulation (before 130.5 ± 81.7 / after 166.5 ± 100.8). End of simulation until 10 min recovery: (before 64.4 ± 47.5 / after 75.3 ± 47.1). Recovery from 10 min to 30 min: (before 105.8 ± 73.2 / after 131.3 ± 77.5). Rest until 30 min recovery: (before 346.0 ± 219.0 / after 441.3 ± 247.6).	Objective performance: open and directed questions (before 3.2±2.0 / after 5.2 ± 3.5). Support: recognition and empathy (before 23.3 ± 14.4 / after 27.1 ± 15.2). Information: procedural information, negotiation and other information (before 63.4 ± 22.5 / after 45.4 ± 24.2).	Medicine.	Cortisol was higher in the pre-simulation and lower in the post-simulation. Physiological levels remain high even when students are training more effectively.
	CG: non trained residents.	CG: 48			Rest until end of preparation: (before 31.6 ± 14.0 / after 32.4 ± 18.4). End of preparation to end of simulation: (before 119.4 ± 57.2 / after 121.2 ± 66.5). End of simulation until 10 min recovery (before 56.4 ± 33.2 / after 54.9 ± 28.6). Recovery from 10 min to 30 min: (before 95.4 ± 53.4 / after 99.1 ± 48.2). Rest until 30 min recovery: (before 312.1 ± 152.3 / after 307.5 ± 159.4).	Objective performance: open and directed questions (before 3.3 ± 2.7 / after 2.8 ± 2.5). Support: recognition and empathy (before 24.2 ± 17.3 / after 22.3 ± 14.0). Information: procedural information, bargaining and other information (before 64.8 ± 29.0 / after 64.9 ± 28.5).		

2012, Harvey et al., Canada^(10)^.	IG: High-stress simulation (HS).	IG: 7	To examine the stress responses of residents during high and low stress simulated trauma resuscitations.	Salivary cortisol	+1,56 nmol/L(1,09).	Checklist: 43.6% (±3.2). GRS: 59.2% (±5.4). ANTS: 66.8% (±4.6). FHT: 60.5% (±3.75).	Medicine.	High-stress trauma simulation produced high cortisol levels and objective measures of stress and lower resident performance.
	CG: Simulação de baixo estresse (LS).	CG: 6			–1,23 nmol/L (1,21).	Checklist: 48.0% (±2.6). GRS: 60.8% (±3.6). ANTS: 70.3% (±3.3). FHT: 68.6% (±2.8).		

2012, Finan et al., Canada^(6)^.	IG: High fidelity simulation. CG: Low fidelity simulation.	IG: 8 CG: 8	To compare the effects of HFS versus LFS technology on objective and subjective measures of stress in a group of neonatology trainees.	Salivary cortisol	Mean baseline level of 7.4 ± 3.7; peak of 14.9 ± 8.7 after the simulated event. Median change in cortisol over the simulations: 6.28 [1.94, 8.91], with no differences between the two groups (p < 0.001).	The mean overall performance score (NRP) was (75.85% ± 10.8) and the mean (ANTS) score was (2.86 ± 0.50). When comparing the groups, there was no significant difference in performance as measured by the (NRP) score (78.2% ± 11.7) (LFS) versus (HFS) (72.7% ± 9, p = 0.17).	Medicine.	The use of HFS and LFS technology resulted in an increase in subjective and objective stress measures. High-fidelity simulation offered no additional benefits in terms of stress modification.

2017, Bong et al., Singapore^(26)^.	IG: Training based on high-fidelity simulation (HFS).	IG:13	To explore the differences between stress levels and non-technical performance among trainees.	Salivary cortisol	Session 1: 0.12 µg/dL (0,05, 0,19) 0.05. Session 2: 0.07 µg/dL (0.001, 0.15) 0.03. Session 3: 0.09 µg/dL (0.01, 0.16) 0.05.	Session 1: 36.7 (34.6, 38.9). Session 2: 39.6 (37.5, 41.7). Session 3: 40.0 (37.9, 42.1).	Medicine, Nursing.	The observers of the immersive simulation-based training achieved an equivalent level of non-technical performance, while experiencing less stress than those repeatedly trained in the hot-seat.
	CG: Interactive educational training session.	CG: 14			Session 1: –0.06 µg/dL (–0.13, 0.01) 0.02. Session 2: 0.01 µg/dL (–0.06, 0.08) –0.01. Session 3: 0.14 µg/dL (0.07, 0.22) 0.15.	Session 3: 39,4 (37.4, 41.5).		

2009, Muller et al., Germany^(27)^.	IG: Crew resource management training (CRM).	IG: 17 CG: 12	Compare the effects of stress and performance in simulated resource management training and classical simulation training.	Salivary cortisol	Before: (12.5 ± 8.4). Immediately after: (15.9 ± 10.2). 15 minutes after: (19.5 ± 12.0).	Task management: pre (12.0 ± 4.3) / post (15.3 ± 3.4). Decision–making: pre (6.7 ± 2.4) / post (8.9 ± 2.3). General Performance: pre: (5.9 ± 2.0) / post: 7.4 ± 1.5)	Medicine.	Pre-simulation cortisol and salivary amylase values were higher compared to post-simulation. Cortisol concentration and salivary amylase activity showed a significant increase during the test scenarios.
	CG: Classical simulation training (MED).	CG: 12			Before: (5.2 ± 2.7). Immediately after: (8.0 ± 6.0). 15 minutes after: (13.2 ± 12.2).	Task management: pre (12.0 ± 3.8) / post (14.7 ± 4.9). Decision–making: pre (6.7 ± 2.4) / post (8.6 ± 2.7). General Performance: pre (5.6 ± 1.6) / post: 6.6 ± 1.6).		

2016, Lee et al., South Korea^(28)^.	IG: Childbirth simulation.	IG:12	To investigate whether the emotional state, measured by salivary cortisol levels of final year nursing students, could predict their acquisition of knowledge and self-confidence.	Salivary cortisol	Conhecimento: B: 0.15 t: 2.63 (p = 0.17). Self–confidence: B: 0.10 t: 0.31 (p = .7590).	Conhecimento: 2.00 (1.13). Autoconfiança: 6.17 (6.00).	Nursing.	The students who took part in the childbirth simulation gained more knowledge and confidence and this was associated with higher cortisol levels.
	CG: Watching a video of the normal childbirth process.	CG: 11				Knowledge: 0.18 (1.08). Self–confidence: 0.73 (5.31).		

2013, Pottier et al., Belgium^(29)^.	IG: Low stress consultation (LS)	IG: 20	To evaluate the impact of subjective and physiological stress on the decision-making and communication skills of students in the context of outpatient consultations.	Salivary cortisol	Day 1: change of pre–scenario: 1.75 (1.01). Day 2: change from pre–scenario: 0.10 (0.73).	Overall communication: baseline: 64.9% (21.7) / study day: 69.7% (12.9). Clinical skills: baseline: 62.8% (15.0) / study day: 64.8% (10.8).	Medicine.	The study showed negative correlations between clinical reasoning and stress. Students who exhibited higher levels of subjective and physiological stress obtained fewer arguments for differential diagnoses.
	CG: High-stress consultation (HS)	CG: 21			Day 1: change from pre–scenario: 1.51 (0.99). Day 2: change from pre–scenario: 3.63 (0.71).	Overall communication score: baseline: 55.7% (18.4) / study day: 68.2% (14.1). Clinical skills: baseline: 58.0% (11.5) / study day: 60.9% (10.9).		

IG: Intervention Group; CG: Control Group; SIM: High Fidelity Simulation; SP: Standardized Patients; HFS: High Fidelity Simulation, LFS: Low Fidelity Simulation; CS: Cortisol; IQR: Interquartile Range; CI: Confidence Interval; CHECKLIST: Institutional Performance Assessment Verification Tool; GRS: Global Rating Scale; ANTS: Anesthesiologist Non-Technical Skills Assessment Tool; FHT: Standardized Trauma History Form; NRP: Advanced Megacode Assessment.

### Risk of Bias

The critical appraisal tool used was the Collaboration Risk of Bias Tool (RoB 2. tool)^(21)^. This is an appraisal tool to assess the risk of bias of the included studies, which makes it possible to evaluate the process of generating sequences, allocation concealment, blinding of participants, personnel and evaluators, incomplete results data and selective reporting of randomized clinical trials. Two researchers independently assessed the quality of each study, and any disagreement was resolved by the third researcher.

## RESULTS

**Figure 1:**
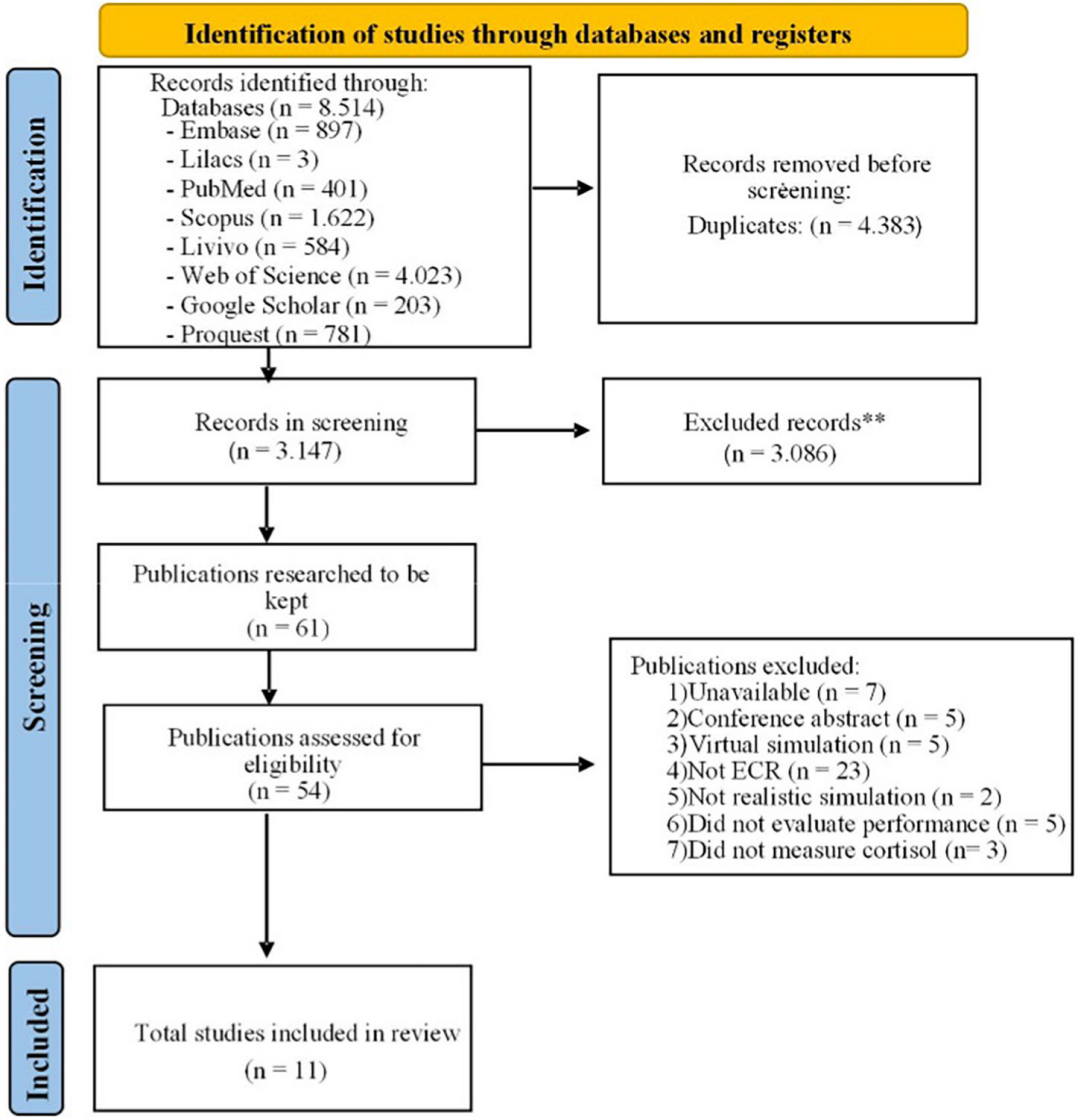
Flowchart of the literature search process and study selection criteria (adapted from the Preferred Reporting Items for Systematic Reviews and Meta-Analyses – PRISMA 2020), Brasília, DF, Brazil, 2024.

The searches carried out in the eight electronic databases used in this systematic review retrieved 8.514 articles. After removing duplicates, 3147 articles were available for screening. From this, 61 studies were selected for full reading, of which 11 met all the eligibility criteria for this review. The process of searching and selecting the studies is detailed in Figure 1.

All the included studies were RCTs and used the SC as the standard for measuring stress. The studies mentioned measures that could influence cortisol reactivation. Among these measures were progesterone and estrogen in salivary samples^(22)^, general infections, diseases of the immune system, endocrine or metabolic diseases, allergies, medications in use (except oral contraceptives), history of neurological disease or psychiatric disorder, smoking, regular consumption of alcohol and drugs, practicing strenuous sports, individuals preparing for exams six weeks before the study, as well as pregnant women, and those who had undergone an examination in the last six weeks^(9)^, medical conditions involving the hypothalamic-pituitary-adrenal axis, recent exposure to exogenous glucocorticoids, mineralocorticoids, anabolic steroids^(23)^, endocrine diseases, pregnancy, medications such as inhaled and systemic steroids and beta-blockers^(24)^.

In addition, some guidelines were given to study participants, such as refraining from eating, drinking caffeinated liquids and fruit juices, smoking and sleeping 4 hours before taking part in the study, not drinking alcohol or doing any heavy activity 24 hours before each experimental session^(9)^, not consuming food, alcohol and/or nicotine half an hour before the evaluation and not exercising 24 hours before collection^(25)^, refraining from eating and drinking for 1 hour before the study period^(9,10,22)^ and rinsing the mouth with water 10 minutes before sample collection^(22)^.

The studies were published between 2009 and 2017^(6,9,10,22–29)^, carried out in the United States^(23)^, Canada^(6,10,22,24)^, Singapore^(26)^, Germany^(9,27)^, South Korea^(28)^ and Belgium^(25,29)^.

Ten additional studies used some kind of simulator^(6,9,10,22–24,26–28)^ and another two studies used standardized patients^(25,29)^.

The participants were nursing^(28)^ and medical^(9,23,29)^ students, Intensive Care Unit (ICU) physicians^(27)^, medical and emergency residents^(10)^, pediatric residents^(22)^, neonatal and perinatal residents^(6)^, oncology residents^(25)^, anesthesiology residents^(26)^, and ICU residents^(24)^. Nine studies were funded^(9,10,22-27,29)^.

The duration of the simulation sessions varied between the included studies, being 10 minutes^(6,27)^, 15 minutes^(9,28)^, 20 minutes^(25)^, or 12 to 15 minutes^(26)^.

Technical performance was measured objectively using a scenario-related checklist^(10,23,27,29)^, European Resuscitation Council guidelines^(9)^, advanced megacode evaluation (NRP)^(22)^, or using the Korean nursing licensing exam^(28)^. In addition, one study assessed performance subjectively, using a self-reported questionnaire^(25)^.

Non-technical skills were assessed using the Anesthesiologist Non-Technical Skills (ANTS) assessment tool^(6,10,23,26,27)^, the Ottawa Global Crisis Resource Management Scale (Ottawa GRS)^(24)^ and a likert scale for cognitive assessment^(10)^.

### Data Synthesis

**Figure 2:**
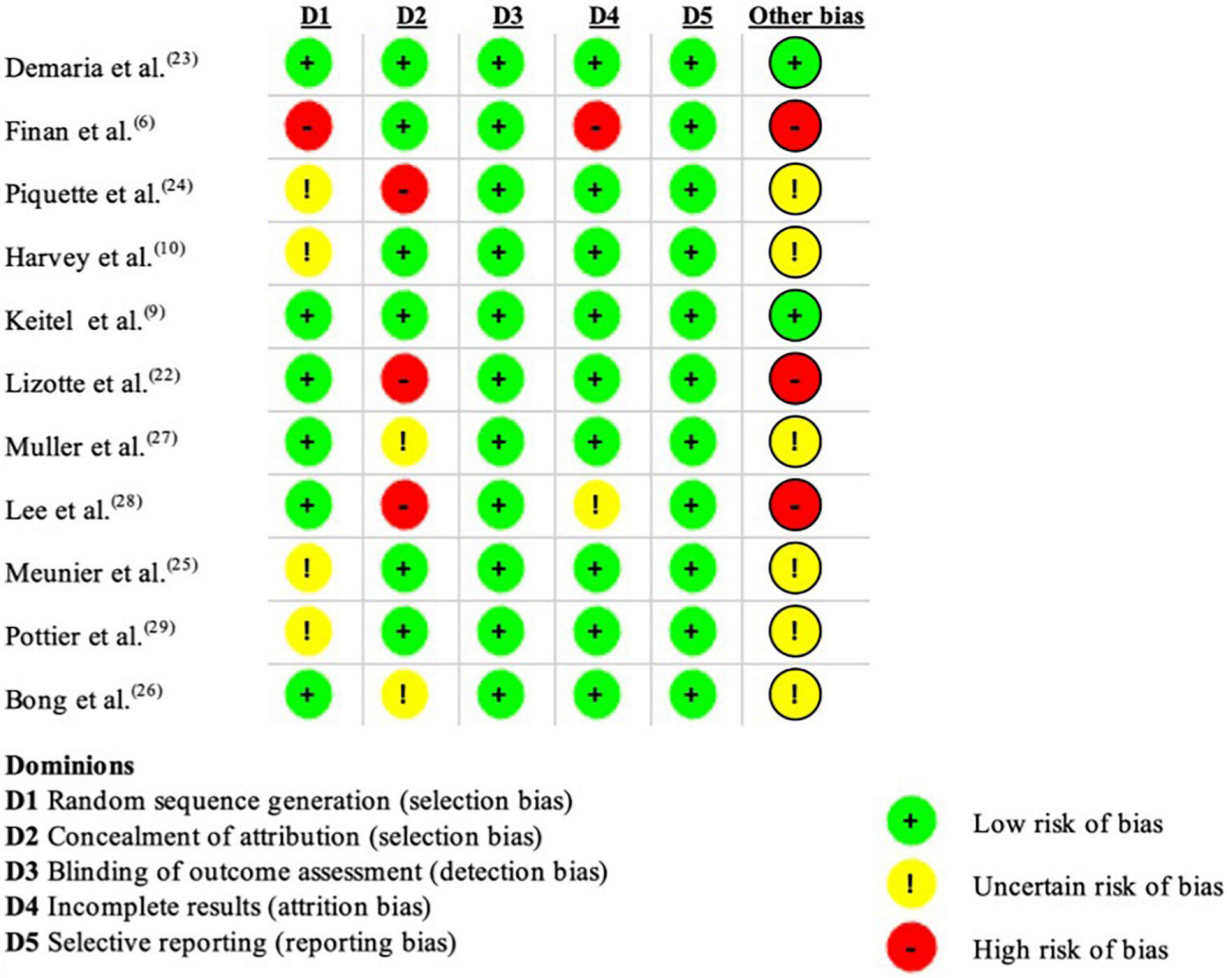
Methodological evaluation of included studies based on the Cochrane tool^(21)^. Brasília, DF, Brazil, 2024.

All the studies analyzed assessed participants’ stress using physiological measures. Other measures were also used to assess stress, such as psychological and self-reported measures. The risk of bias of the studies was assessed as low, high or unclear (Figure 2).

No association was found between performance and stress in low-fidelity scenarios compared to high-fidelity scenarios (p = 0.17)^(6)^, nor in resuscitation scenarios (p = .098)^(24)^. However, in other studies, participants performed significantly worse in the high-stress condition (p < 0.012), indicating that the high-stress situation can be seen as a threat, leading to impaired performance^(10)^. In simulated outpatient consultations, medical students experienced deleterious effects on clinical reasoning in high-stress conditions^(29)^.

In postpartum neonatal resuscitation scenarios with simulated death (p = 0.23) or survival (p = 0.33), performance was similar in 1st and 2nd year medical residents compared to the performance of 3rd and 4th year residents^(22)^. In another study, there was no statistically significant difference between medical students in the death group compared to survival (p = 0.89)^(23)^.

Additionally, in a high-fidelity emergency scenario compared to laboratory stress, cortisol increased in both conditions, but no association was found between stress and performance during the simulation (p = 0.631)^(9)^. In another emergency simulation study, after one day’s training, participants produced significant stress and performance improved (p < 0.01)^(27)^.

Higher cortisol levels in nursing students were associated with greater knowledge attainment in a childbirth training and simulation scenario (p < 0.001)^(28)^. Communication skills training has an effect on physiological arousal. After simulated training, cortisol levels increased significantly compared to the control group, improving self-efficacy and communication skills (p = 0.026)^(25)^. In another study, performance was similar in non-technical skills between the active versus observer roles^(26)^.

The risk of bias was assessed per study. In relation to the selected studies, one study^(6)^ presented a high risk of bias in two domains because there was a difference between the sex of the participants (more female participants) and the groups had “clues” provided by the facilitators in the scenarios, but the clues may have been less obvious in one of the groups, which may have created a discrepancy in the participants’ understanding. In another study^(24)^, there was a low bias risk and an uncertain bias risk, the study population was heterogeneous (participants from various levels of training and specialties) and the person supervising the simulation sessions gave feedback to the residents. This may have been perceived as a source of stress and influenced the results. In others, there was an uncertain bias risk, the participants were of various levels of training^(10)^; there was insufficient information about the randomization process^(25,29)^; one participant was excluded because his cortisol was 10 times higher, no sensitivity test was reported in the study^(26)^; the allocation of participants was by random draw^(27)^, and another factor that produced a high risk of bias, was that approximately 29% of the participants who consented to the study did not complete the simulation sessions, resulting in a loss of follow-up of the participants. We don’t know if all the events of interest were adequately captured and correctly scored^(22)^ and voluntary selection has been shown to be a risk of bias^(28)^.

## DISCUSSION

This is a systematic review of the available evidence on cortisol levels and participant performance in realistic simulations, evaluated in 11 randomized clinical trials.

High-fidelity simulation has been shown to be significantly stressful, as evidenced by increased cortisol levels^(9,10,22-27,29)^. In other studies, high- and low-fidelity scenarios triggered significant stress responses^(6,24,30,31)^, suggesting that high-fidelity simulation is not superior to low-fidelity^(6)^. In addition, in the medium-fidelity simulation, cortisol increased significantly^(28)^.

With regard to trends in studies over time, it can be seen that there has been an increase in the number of studies comparing high-fidelity and low-fidelity, as well as high-stress and low-stress simulation, the emerging field being medicine. In terms of sub-groups, the study population was mostly made up of residents from different areas of medicine and medical students.

In some studies, no correlation was found between physiological markers of stress and the participants’ performance^(6,9,22–24,26)^. In this context, it is essential to recognize that the absence of this correlation between stress and performance in certain studies can be attributed to various conditions. For example, the heterogeneity of the sample, made up of participants with different levels of training and different specialties, who may vary in their performance due to their different backgrounds. In addition, the varied nature of the stresses used in the studies may have triggered divergent physiological and psychological responses among the participants, resulting in different effects on performance and, consequently, significantly impacting the results^(24)^.

Furthermore, it is important to note that the non-completion of the simulation sessions by some participants and the non-blinding of the reviewers to the nature of the study, and to the identification of the participants and the scenario are additional factors that may have introduced potential biases in the assessment of performance^(21)^. The small number of participants and larger representation of female members may influence the external validity of the findings^(6)^, since stress responses and adaptation mechanisms may vary between genders. Another point to be made is that the clues provided by the facilitators to the participants in the simulation scenario may have been less obvious between the groups, creating potential discrepancies between the participants’ understanding of the patient’s underlying physiological state^(6)^. These methodological issues need to be properly considered when interpreting the results in order to ensure a more comprehensive and accurate understanding of the relationships between stress and performance in the specific contexts addressed.

Nevertheless, other studies have identified that high levels of acute stress can critically impair medical decision-making^(10)^ and have been associated with changes in clinical reasoning, causing doctors to be less able to establish diagnoses^(29)^.

However, in other studies, stress has been shown to be beneficial to participants’ performance. High levels of stress resulted in improved clinical and non-technical performance^(27)^, basic knowledge^(28)^ and improved communication of bad news^(25)^.

Responses to stress, determined by the individual’s perception of demands and resources^(10)^ are considered ideal for detecting warning signs and mitigating responses. Coping skills can be improved to maintain allostasis, while ineffective coping, related to changes in the regulation and responsiveness of the hypothalamic-pituitary-adrenal (HPA) axis and release of the hormone cortisol, are associated with impaired performance^(32,33)^.

The impact of acute stress on performance is still debated^(34)^. Failure to fully understand the impact of student stress on training performance involves the danger of impairing learning and the acquisition of clinical skills during training, and may result in individuals being inadequately prepared to deal with real situations^(34)^. It is already known that performance in high acuity situations can be improved or impaired, depending on the perception of the demand and resources of the individuals^(35)^ assuming that performance increases with the level of stress up to a certain limit beyond which performance decreases, suggesting that stress puts the person at a point of cognitive deficit^(36)^.

Despite the inherent importance of simulation teaching, a lack of experience and emotional mastery can trigger a stress reaction, potentially impacting student performance^(37)^. The anticipation of critical situations and the perception of being watched induce activation of the autonomic nervous system (ANS) and the hypothalamic-pituitary-adrenal (HPA) axis associated with higher cortical functions^(38)^. The sympathetic response of the autonomic system leads to an increase in blood pressure, heart rate, skin temperature and anaerobic metabolism, while activation of the HPA results in increased secretion of cortisol into the blood, which is then diffused into saliva over a period of minutes^(34,38,39)^. The increase in cortisol levels has an impact on brain regions closely related to cognitive processes, including the amygdala, hippocampus and prefrontal cortex^(34)^.

For these reasons, stress management training has been shown to be effective in reducing stress^(40)^, with positive effects not only on stress indicators, but also on performance^(41–43)^.

Acute stress can be a risk factor for diagnostic errors^(29)^ and impaired patient safety^(10)^. On the other hand, it can improve clinical performance and non-technical skills^(27)^, can prepare residents to deal with death^(22)^, can improve advanced life support skills^(23)^, knowledge retention and consolidation^(26)^, as well as offering greater clinical skills in deliveries^(28)^ and verbal communication^(25)^.

The effects of stress depend on a number of factors, including gender, previous experience, personality traits, psychological assessment, assigned role and team attribution^(10,33)^. However, it is still difficult to know the precise origin of stress^(44)^.

With regard to additional stressors, the presence of observers, filming, team dynamics and the perception of evaluation may have influenced stress^(6,26,31)^. However, in a simulation of laparoscopic surgery, noise did not cause changes in stress levels^(45)^. Another study^(46)^ showed that distractions such as telephone calls during the simulation caused changes in physiological parameters. In the study by Piquette and colleagues^(24)^, these stressors appeared to be weak enough to provoke a stress response among residents used to a hectic environment. Therefore, the stressors themselves may not lead to impaired performance^(10)^. Performance can be impacted by mental stress, with the addition of elements to the scenario that put the cognitive system at risk of overload. In this context, in stressful episodes, attention can be directed exclusively to specific tasks, resulting in the possible neglect of other potentially relevant information^(18)^. Therefore, caution is needed when designing scenarios, taking care to eliminate as many potential distractions as possible from these clinical environments^(10)^, being aware of the mechanism of stress and offering resources for its management^(46)^.

Regarding the exclusion of participants in studies due to conditions that could influence cortisol levels, this factor can have important implications for the generalizability of the results and can introduce potential biases into the conclusions. By restricting participation on the basis of cortisol-related factors, such as medical conditions or the use of medications that affect hormone regulation, there is a risk of limiting the representativeness of the sample. On the other hand, selective exclusion of participants can result in a more homogeneous sample, underestimating or overestimating the effects of cortisol. In this way, researchers should be aware of all the conditions that can modulate cortisol, reducing the risk of confounding bias.

As for the limitations of the studies, it is important to highlight the blinding bias of the participants and evaluators and the lack of a validated performance evaluation tool suitable for the scenarios, making it impossible to generalize the results and the small sample size as it does not provide the necessary statistical power. Some studies had participants with different levels of experience, training and specialty in their sample, and the sample population was chosen for convenience or included on a voluntary basis, which can have a negative impact on the level of evidence in the studies. Furthermore, most of the studies did not address the participants’ previous experience with the simulation.

In relation to the limitations inherent to this review, we would highlight the lack of a meta-analysis due to the heterogeneity of the studies included in relation to method, sample design and statistical analysis. There were also challenges related to access to data in some studies and, unfortunately, when trying to contact the author responsible for the article, we were unsuccessful. This difficulty may have resulted in the possible loss of relevant information that could have contributed to the inclusion and understanding of this review.

The review presented here has allowed us to expand our knowledge of the association between cortisol levels and performance in clinical simulation, highlighting the complexity of the interactions between the endocrine system and performance in simulated environments. The results could be essential for teachers and professionals working with clinical simulation, the studies should take into account the participant’s stress level and the conditions that modulate stress, since cortisol regulation can play a crucial role in the participant’s adaptation and performance. Furthermore, they need to be careful when designing a clinical scenario, knowing the factors that affect performance can contribute to improving clinical practice and enhancing the quality of health care. In addition, stress management must be taken into account so that the simulation is not a trauma, but a form of learning for the student.

## CONCLUSION

This is the first systematic review on the impact of cortisol on performance in simulations, to the best of our knowledge. As cortisol levels change, participants’ performance changes, either in a detrimental or beneficial way. However, in other studies there was no correlation between stress and performance, which may not have been due to methodological issues. It is clear that there is a lack of robust scientific evidence in this area, highlighting the urgent need for more careful and well-designed research. With regard to the research gap, it is not known to what extent stress can be beneficial or detrimental to performance and whether this variation is changeable according to the level of difficulty of the scenario or stressors in the simulation scenario.

With regard to future studies, it is suggested that well-designed randomized clinical trials be carried out to reduce the risk of bias and that they cover a wide range of fidelities, from low to high fidelity trials that assess causality between exposure and outcome, significantly increasing the sample size and having a distinct population in their sample.
