# Supplementary material for: Association between cortisol levels and performance in clinical simulation: a systematic review
Source: Rev Esc Enferm USP. 2024 Jul 26;58:e20230279. doi: 10.1590/1980-220X-REEUSP-2023-0279en (PMC11277686; doi:10.1590/1980-220X-REEUSP-2023-0279en)
Supplement: Translation

# Translation: Associação entre nível de cortisol e desempenho em simulação clínica: revisão sistemática

**DOI:** 10.1590/1980-220X-REEUSP-2023-0279pt

**Authors:** Jackson Gois Teixeira, Lucas Tomaz Benigno Lima, Elaine Carvalho Cunha, Flavia Oliveira de Almeida Marques da Cruz, Karen Karoline Gouveia Carneiro, Laiane Medeiros Ribeiro, Guilherme da Costa Brasil

**Affiliations:** 1 Centro Universitário do Distrito Federal, Departamento de Enfermagem, Brasília, DF, Brasil.; 2 Universidade de Brasília, Programa de Pós-Graduação em Enfermagem, Brasília, DF, Brasil.; 3 Centro Universitário do Planalto Central Apparecido dos Santos, Departamento de Enfermagem, Brasília, DF, Brasil.; 4 Universidade de Brasília, Departamento de Enfermagem, Brasília, DF, Brasil.

**Keywords:** Avaliação de Desempenho Profissional, Estresse Psicológico, Hidrocortisona, Treinamento por Simulação, Revisão Sistemática

## Abstract

**Objetivo:**

Identificar como o estresse mensurado por cortisol salivar durante a educação baseada em simulação clínica ou simulação e outro método de ensino, tem impacto no desempenho.

**Método:**

Revisão sistemática da associação do cortisol com o desempenho em simulações. Foram utilizadas as seguintes bases de dados: PubMed, LIVIVO, Scopus, EMBASE, Literatura Latino-Americana e do Caribe em Ciências da Saúde (LILACS) e Web of Science. Pesquisa adicional de literatura cinzenta foi realizada no Google Scholar e no Proquest. As buscas ocorreram em 20 de março de 2023. O risco de viés dos ensaios clínicos randomizados, foi avaliado pela ferramenta Cochrane Collaboration Risk of Bias Tool (RoB 2). Foram critérios de inclusão: estudos de simulação com coleta de cortisol salivar e avaliação de desempenho, publicados em qualquer período em português, inglês e espanhol.

**Resultados:**

11 estudos foram incluídos, os quais mensuraram o estresse utilizando o cortisol salivar e analisados através da síntese descritiva e da análise qualitativa.

**Conclusão:**

Alguns estudos mostraram haver relação entre estresse e desempenho, podendo ser benéfico ou prejudicial ao participante. Porém, outros estudos não apresentaram essa correlação que pode não ter ocorrido por questões metodológicas.

## INTRODUÇÃO

As simulações em saúde tornaram-se um método de treinamento explorado em laboratórios de ensino e centros de simulação, com a finalidade de desenvolver habilidades técnicas e não técnicas, trazendo benefícios no processo de aprendizagem e contribuindo para a formação e aprimoramento profissional^(1,2)^.

A simulação realística já foi descrita como uma experiência estressora^(3)^. O estresse está associado a impactos cognitivos negativos, como diminuição da concentração, degradação da memória, aumento de erros e retardamento na resposta a estímulos^(4)^. Contudo, até um ponto específico, o estresse pode aprimorar a concentração na tarefa, direcionar o foco na comunicação e contribuir para a resolução de problemas^(5)^. Ademais, as circunstâncias enxergadas como ameaçadoras tendem a desencadear emoções negativas, ao passo que avaliações de desafio estão correlacionadas a respostas emocionais mais positivas^(6)^.

Adicionalmente, em um ensaio clínico randomizado que investigou a adição de estressores emocionais em uma simulação, os participantes foram capazes de lembrar os eventos dos cenários que falharam, indicando que o estresse emocional pode melhorar a capacidade de lembrar essas memórias^(7)^. Desse modo, os níveis moderados de estresse são essenciais para a eficácia no aprendizado ativo do aluno^(8)^.

Entretanto, o sentido causal da relação permanece indeterminada: será que o desempenho elevado está associado a uma menor experiência de estresse, ou a presença constante do estresse em um indivíduo está associada a uma menor tendência a erros^(9)^. Além disso, não se sabe ao certo se os estressores adicionais podem trazer algum prejuízo ao desempenho, uma vez que as limitações de desempenho podem acarretar efeitos imediatos na qualidade da assistência prestada aos pacientes^(10)^.

A literatura define o estresse como um estado de divergência entre as demandas percebidas, as reações do indivíduo e a capacidade de adaptação aos fatores estressores^(3)^ e está intimamente ligado ás emoções, envolvendo respostas emocionais e fisiológicas a um estressor^(11)^. Ainda mais, condições estressantes desencadeiam a ativação dos sistemas endócrino, nervoso e imunológico, fenômeno amplamente reconhecido como resposta ao estresse^(12)^.

No corpo humano, encontramos marcadores bioquímicos de estresse. O cortisol é um hormônio do estresse produzido no córtex adrenal, e sua concentração na saliva está fortemente correlacionada com sua concentração no plasma sanguíneo^(3)^. Os níveis de cortisol salivar têm sido usados extensivamente como uma medida objetiva de estresse em simulação, tornando-se um ensaio ideal para pesquisa^(13)^. Agindo como um marcador biológico, os níveis de cortisol aumentam em resposta ao estresse e às inúmeras mudanças do ambiente de simulação^(8)^.

Inicialmente, estudos observacionais, identificaram elevações nos níveis de cortisol dos participantes^(14-17)^. Contudo, em uma revisão sistemática, foi possível evidenciar que o estresse experimentado em uma simulação ainda é indefinido^(18)^.

Dessa forma, é importante sintetizar por meio de uma revisão sistemática de estudos de intervenção pela análise de grupos qual é a relação do cortisol com o desempenho do participante visto que diante das diferentes experiências e percepções nas simulações, os participantes podem apresentar respostas fisiológicas distintas.

Assim, o objetivo desse estudo foi identificar como o estresse mensurado por cortisol salivar durante a educação baseada em simulação clínica ou simulação e outro método de ensino, tem impacto no desempenho.

## MÉTODO

### Registro e Protocolo

Trata-se de uma revisão sistemática da associação da simulação nos níveis de cortisol e desempenho, conduzida de acordo com as recomendações da Cochrane Collaboration^(19)^ e descrita segundo o *Preferred Reporting Items for Systematic Reviews and Meta-Analyses* – PRISMA^(20)^. O protocolo foi registrado no *International Prospective Register of Systematic Reviews* sob o número CRD42022319886.

### Desenvolvimento da Pergunta de Pesquisa

A pergunta foi norteada pela estratégia PICO, considerando-se “P” (paciente ou problema) estudantes ou profissionais de saúde; “I” (intervenção) simulação realística; “C” (controle) não foi aplicado, e “O” (resultado ou desfecho) como associação entre cortisol e desempenho. Desse modo, a pergunta norteadora foi: Qual a associação entre nível de cortisol salivar e desempenho de participantes em educação baseada em simulação?

### Critérios de Elegibilidade

Nesta revisão sistemática, foram incluídos ensaios clínicos randomizados (ECR) que avaliaram o estresse por meio do cortisol salivar (CS) nos seguintes contextos: (a) simulações realísticas realizadas com estudantes da (medicina, enfermagem); (b) simulações para treinamento de profissionais médicos residentes; (c) simulações que incluíam profissionais de outras áreas da saúde; (d) simulações dentro dos laboratórios institucionais – cenário hospitalar; e (e) simulação de baixa, média e alta fidelidade.

Os estudos foram excluídos pelos seguintes critérios: (a) indisponibilidade (b) resumo de conferência (c) simulação virtual; (d) não ser ECR; (e) não ser desenvolvido no contexto de simulação realística; (f) avaliar a alfa amilase; (g) não avaliar o desempenho; (h) avaliar a ansiedade.

### Bases de Dados e Estratégia de Busca

A busca foi executada nas seguintes bases eletrônicas de dados: PubMed, LIVIVO, Scopus, EMBASE, Literatura Latino-Americana e do Caribe em Ciências da Saúde (LILACS), Web of Science (Índice de Citação em Ciências Sociais). Pesquisa adicional de literatura cinza foi realizada no Google Scholar e no Proquest. A lista das referências dos estudos selecionados foi analisada manualmente para identificar aquelas potencialmente relevantes que poderiam ter sido perdidas nas pesquisas eletrônicas nas bases de dados. As referências duplicadas foram removidas com uso do Rayyan^®^.

### Estratégia de Pesquisa

Os termos de pesquisa foram adaptados para uso nas diferentes bases eletrônicas de dados, combinados com filtros específicos para ensaios controlados quando disponíveis. Foram selecionados estudos nos idiomas português, inglês e espanhol, e sem corte temporal delimitado para abranger um amplo espectro de publicações nacionais e internacionais. Todas as buscas nas bases eletrônicas de dados foram realizadas no dia 20 março de 2023, cujas estratégias estão no Quadro 1.

**Quadro 1:** Estratégias de busca nas bases eletrônicas de dados – Brasília, DF, Brasil, 2024.

Bases de dados	Palavras-chave
**PubMed**	#1 = (“Patient Simulation”[MeSH] OR “Simulation Training”[MeSH] OR “Simulation Training/methods”[MeSH]) OR “High Fidelity Simulation”[All Fields] OR “High Fidelity Simulation Training”[Mesh]) #2 = (“Stress, Psychological”[MeSH] OR “Stress, Physiological”[MeSH] OR “Stress Response”[All Fields] OR “Hydrocortisone”[MeSH] OR Cortisol[All Fields] OR “Salivary Cortisol”[All Fields] OR “Saliva/chemistry”[MeSH]) #3 = #1 AND #2
**LILACS**	(“Patient Simulation” OR “Simulation Training”) AND (“stress, psychological” OR “salivary cortisol”)
**ĹIVIVO**	(“Patient Simulation” OR “Simulation Training” OR “High Fidelity Simulation” OR “High Fidelity Simulation Training” OR “High-Fidelity Manikin” OR Simulation) AND (“Stress, Psychological” OR “Stress, Physiological” OR “Stress Response” OR “Hydrocortisone” OR Cortisol OR “Salivary Cortisol”)
**SCOPUS**	(ALL (“Patient Simulation” OR “Simulation Training” OR “High Fidelity Simulation” OR “High Fidelity Simulation Training” OR “High-Fidelity Manikin” OR simulation) AND TITLE-ABS-KEY (cortisol OR “Salivary Cortisol”) )
**Web of Science**	TS=((“Patient Simulation” OR “Simulation Training” OR “High Fidelity Simulation” OR “High Fidelity Simulation Training” OR “High-Fidelity Manikin” OR Simulation) AND TS=(“Stress, Psychological” OR “Stress, Physiological” OR “Stress Response” OR “Hydrocortisone” OR Cortisol OR “Salivary Cortisol”)
**EMBASE**	#1 = (“patient simulation”/exp OR “patient simulator”/exp OR “simulation training”/exp OR “high fidelity simulation training”/exp OR “high fidelity simulation”) #2 = (“psychological stress”/exp OR “Stress, Physiological” OR ‘mental stress’/exp “Stress Response” OR hydrocortisone/exp OR cortisol OR “salivary cortisol”) #3 = #1 AND #2
**Google Scholar**	(“Patient Simulation” OR “ Simulation Training” OR simulation) AND (“psychological stress” OR “cortisol”) Where my words occur: anywhere in the article 100 most relevant hits (10 pages)
**Proquest**	(“Patient Simulation” OR “Simulation Training” OR “Simulation Training/methods” OR “High Fidelity Simulation” OR “High Fidelity Simulation Training”) AND (“Stress, Psychological” OR “Stress, Physiological” OR “Stress Response” OR “Hydrocortisone” OR Cortisol OR “Salivary Cortisol” OR “Saliva/chemistry”)

### Processo de Seleção

A seleção dos estudos aconteceu em duas fases, por meio do aplicativo on-line Rayyan^®^ (Qatar Computing Research Institute), um programa que agiliza a triagem inicial dos estudos por meio de um processo de semiautomação, que garante a confiabilidade da seleção. Na primeira fase, dois pesquisadores examinaram, de forma independente, os títulos e resumos de todos os estudos recuperados nas bases de dados e identificaram aqueles que atendiam aos critérios de inclusão. Na segunda fase, os mesmos pesquisadores leram, também de forma independente, o texto completo de todos os estudos selecionados e excluíram aqueles que não atenderam aos critérios de inclusão. Qualquer divergência nessa fase seria resolvida por discussão entre os pesquisadores e um especialista, o qual também avaliaria de forma independente o estudo em texto completo.

### Processo de Coleta de Dados

Dois pesquisadores extraíram os dados dos estudos incluídos nesta revisão sistemática de forma independente, por meio de um instrumento de coleta de dados de criação própria. Qualquer divergência foi resolvida por discussão e acordo mútuo. Um terceiro autor estava envolvido quando necessário para tomar uma decisão final.

As variáveis coletadas incluíram: características dos participantes (grupos e amostra); características do estudo (autores, país, ano de publicação, objetivo, delineamento, randomização e critérios de inclusão e exclusão); intervenção (tipo de simulação, simulador, área de conhecimento); coleta (medida do cortisol); e características dos resultados (principais resultados e principais conclusões). Se os dados necessários não estivessem completos, seria feito contato com os autores para obtenção de qualquer informação relevante. A partir desses dados, os resultados dessa revisão sistemática são apresentados de forma descritiva no Tabela 1.

**Tabela 1:** Características gerais dos estudos incluídos de acordo com ano, autor, país, grupos, amostra, objetivo, mensuração do estresse, cortisol, desempenho, área e conclusões - Brasília, DF, Brasil, 2024.

Ano, autor, país	Grupos	N	Características do estudo / objetivo	Mensuração do cortisol	Cortisol	Desempenho	Área	Principais conclusões
2017, Lizotte et al., Canadá^(22)^.	GI: Simulação com morte.	GI: 21	Avaliar o impacto das simulações no estresse e desempenho dos treinandos; tanto durante uma simulação “tradicional” (manequins-sobreviventes) quanto durante a morte simulada.	Cortisol Salivar.	T0: 0,10 µg/dL [IQR 0,07–0,14]. T1: 0,11 µg/dL [IQR 0,10–0,17]. T2: 0,17 µg/dL [IQR 0,13–0,28].	Primeiro cenário: 82 [IQR 78–88] = 0,85. Segundo cenário: 79 [IQR 77–86] = 0,87.	Medicina.	A simulação neonatal causa estresse antes e durante a simulação sem interferir no desempenho. Ter um manequim “morto” durante uma simulação não aumenta o estresse objetivo nem interfere no desempenho.
	GC: Simulação com sobrevida	GC:21			T0: 0,10 µg/dL [IQR 0,06–0,15]. T1:0,15 µg/dL [IQR 0,09–0,22]. T2:0,23 µg/dL [IQR 0,14–0,47].	Primeiro cenário: 83 [IQR 74–89] =0,85. Segundo cenário: 82 [IQR 72–88] = 0,87.		

2011, Keitel et al., Alemanha^(9)^.	GI: Situação de emergência simulada.	34	Avaliar as respostas psicológicas e endócrinas do estresse em simulação realística e a relação desempenho e estresse.	Cortisol Salivar.	–15 min: 0.25µg/dL (–0,25–0,75 IC). 0 min: 0.22 µg/dL (–0.27–0.72 IC). 15 min: 0.26 µg/dL (–0,24–0.76 IC). 30 min: 0.11 µg/dL (–0.39–0.61). 45 min: –0.15 µg/dL (–0.65–0.35 IC). 60 min: –0.25 µg/dL (–0.75–0.25 IC). 75 min: –0.10 µg/dL (–0.60–0.40 IC).	Sem correlação significativa entre aumento do cortisol salivar e desempenho (p = 0,811 e p = 0,631).	Medicina.	A relação positiva entre a resposta ao estresse endócrino em uma situação laboratorial padrão e desempenho em uma situação de emergência simulada indica que alta capacidade de resposta ao estresse pode ser um preditor de bom desempenho.
	GC: Condição de Repouso.		correlacionou–se significativamente com o aumento do cortisol (p = 0,019).					

2016, Demaria et al., Estados Unidos^(23)^.	GI: Simulação com morte.	GI: 13	Descrever a resposta de estresse fisiológico e bioquímica entre simulação com morte e simulação com sobrevida.	Cortisol Salivar.	0,193 µg/dL. O aumento médio de SC foi de 0.053 µg/dL [0.071 a 0.165].	83,3% [75–85,8] = (p = 0,18).	Medicina.	Não houve uma resposta negativa a uma morte simulada do paciente em comparação com a sobrevida simulada. O cortisol salivar aumentou em comparação aos níveis basais, porém não houve diferenças significativas.
	GC: Simulação com sobrevida.	GC: 13			0,159 µg/dL. 0,056 µg/dL [0,033–0,163] não havendo diferença estatística entre os grupos.	75% [64,1–84,2].		

2014, Piquette et al., Canadá^(24)^.	GI: Simulação com cenário de alto estresse.	GI: 26	Explorar os efeitos de estressores externos modificáveis no desempenho clínico simulado de residentes.	Cortisol Salivar.	Pré cenário (–15 min): 7.65 ± 5.19. Pré cenário (–5 min): 9.07 ± 6.39. Pós cenário (0 min): 9.25 ± 7.17. Pós cenário (10 min): 9.71 ± 7.00. Pós cenário (20 min): 8.33 ± 5.08.	4.7 ± 0.9. 72% ± 11%.	Medicina.	Houve respostas significativas de estresse fisiológico e psicológico dos residentes ao passarem por cenários de ressuscitação simulada. Os níveis de cortisol demostraram melhor desempenho nos grupos A.
	GC: Simulação com cenário de baixo estresse.	GC: 28			Pré cenário (–15 min): 7.11 ± 4.50. Pré cenário (–5 min): 8.14 ± 5.11. Pós cenário (0 min): 8.64 ± 6.10. Pós cenário (10 min): 9.20 ± 6.67. Pós cenário (20 min): 7.70 ± 5.69.	4.9 ± 0.8. 70% ± 11%.		

2013, Meunier et al., Bélgica^(25)^.	GI: residentes treinados.	GI: 50	Avaliar o efeito do treinamento de habilidades de comunicação na excitação fisiológica dos residentes durante a comunicação de más notícias.	Cortisol Salivar.	Repouso até final da preparação (antes 32.4 ± 22,0 / pós 44,9 ± 28,0). Final da preparação até final da simulação (antes 130.5 ± 81.7 / pós 166,5 ± 100,8). Final da simulação até recuperação de 10 min: (antes 64.4 ± 47.5 / pós 75.3 ± 47.1). Recuperação de 10 min até recuperação de 30 min: (antes 105.8 ± 73.2 / pós 131.3 ± 77.5). Repouso até recuperação de 30 min: (antes 346.0 ± 219.0 / pós 441.3 ± 247.6).	Desempenho objetivo: perguntas abertas e direcionadas (antes 3.2 ± 2.0 / pós 5.2 ± 3.5). Suporte: reconhecimento e empatia (antes 23.3 ± 14.4 / pós 27.1 ± 15.2). Informação: informação procedimental, negociação e outras informações (antes 63.4 ± 22.5 / pós 45.4 ± 24.2).	Medicina.	Cortisol apresentou-se mais elevado na pré-simulação e menores na pós-simulação. Os níveis fisiológicos se mantêm elevados mesmo quando os alunos realizam o treinamento com maior eficácia.
	GC: residentes não treinados.	GC: 48			Repouso até final da preparação: (antes 31.6 ± 14.0 / pós 32,4 ± 18,4). Final da preparação até final da simulação: (antes 119.4 ± 57.2 / pós 121,2 ± 66,5). Final da simulação até recuperação de 10 min (antes 56.4 ± 33.2 / pós 54,9 ± 28,6). Recuperação de 10 min até recuperação de 30 min: (antes 95.4 ± 53.4 / pós 99.1 ± 48,2). Repouso até recuperação de 30 min: (antes 312.1 ± 152,3 / pós 307.5 ± 159.4).	Desempenho objetivo: perguntas abertas e direcionadas (antes 3.3 ± 2.7 / pós 2.8 ± 2.5). Suporte: reconhecimento e empatia (antes 24.2 ± 17.3 / pós 22.3 ± 14.0). Informação: informação procedimental, negociação e outras informações (antes 64.8 ± 29,0 / pós 64.9 ± 28.5).		

2012, Harvey et al., Canadá^(10)^.	GI: Simulação de alto estresse (HS).	GI: 7	Examinar as respostas ao estresse de residentes durante reanimações de trauma simuladas de alto e baixo estresse.	Cortisol Salivar.	+1,56 nmol/L(1,09).	Checklist: 43.6% (±3.2). GRS: 59.2% (±5.4). ANTS: 66.8% (±4.6). FHT: 60.5% (±3.75).	Medicina.	Simulação de trauma de alto estresse produziu níveis de cortisol e as medidas de objetivas de estresse elevados e menor desempenho dos residentes.
	GC: Simulação de baixo estresse (LS).	GC: 6			–1,23 nmol/L (1,21).	Checklist: 48.0% (±2.6). GRS: 60.8% (±3.6). ANTS: 70.3% (±3.3). FHT: 68.6% (±2.8).		

2012, Finan et al., Canadá^(6)^.	GI: Simulação de alta fidelidade. GC: Simulação de baixa fidelidade.	GI: 8 GC: 8	Comparar os efeitos da HFS versus a tecnologia da LFS nas medidas objetivas e subjetivas de estresse em um grupo de estagiários em neonatologia.	Cortisol Salivar.	Nível basal médio de 7,4 ± 3,7; pico de 14,9 ± 8,7 após o evento simulado. Mudança mediana no cortisol ao longo das simulações: 6,28 [1,94, 8,91], sem diferenças entre os dois grupos (p < 0,001).	A pontuação média geral de desempenho (NRP) foi de (75,85% ± 10,8) e a pontuação (ANTS) média foi de (2,86 ± 0,50). Ao comparar os grupos, não houve diferença significativa no desempenho, conforme medido pela pontuação (NRP) (78,2% ± 11,7) (LFS) versus (HFS) (72,7% ± 9, p = 0.17).	Medicina.	O uso da tecnologia HFS e LFS resultou em aumento das medidas de estresse subjetivo e objetivo. A simulação de alta fidelidade não ofereceu benefícios adicionais em termos de modificação do estresse.

2017, Bong et al., Singapura^(26)^.	GI: Treinamento baseado em simulação de alta fidelidade (HFS).	GI:13	Explorar as diferenças entre os níveis de estresse e o desempenho não-técnico entre estagiários.	Cortisol Salivar.	Sessão 1: 0.12 µg/dL (0,05, 0,19) 0.05. Sessão 2: 0.07 µg/dL (0.001, 0.15) 0.03. Sessão 3: 0.09 µg/dL (0.01, 0.16) 0.05.	Sessão 1: 36.7 (34.6, 38.9). Sessão 2: 39.6 (37.5, 41.7). Sessão 3: 40.0 (37.9, 42.1).	Medicina, Enfermagem.	Os observadores do treinamento baseado em simulação imersiva alcançaram um nível equivalente de desempenho não técnico, ao mesmo tempo em que experimentaram menor estresse do que os treinandos repetidamente no ‘hot-seat’.
	GC: Sessão interativa de formação educacional.	GC: 14			Sessão 1: –0.06 µg/dL (–0.13, 0.01) 0.02. Sessão 2: 0.01 µg/dL (–0.06, 0.08) –0.01. Sessão 3: 0.14 µg/dL (0.07, 0.22) 0.15.	Sessão 3: 39,4 (37.4, 41.5).		

2009, Müller et al., Alemanha^(27)^.	GI: Treinamento de gerenciamento de recurso de tripulação (CRM).	GI: 17 GC: 12	Comparar os efeitos do estresse e desempenho no treinamento simulado de gerenciamento de recurso e treinamento de simulação clássica.	Cortisol Salivar.	Antes: (12.5 ± 8.4). Imediatamente após: (15.9 ± 10.2). 15 minutos após: (19.5 ± 12.0).	Gerenciamento de tarefas: pré (12.0 ± 4.3) / pós (15.3 ± 3.4). Tomada de decisão: pré: 6.7 ± 2.4) / pós 8.9 ± 2.3). Desempenho Geral: pré: (5.9 ± 2.0) / pós: 7.4 ± 1.5)	Medicina.	Os valores de cortisol e amilase salivar pré-simulação foi maior se comparado ao pós-simulação. A concentração de cortisol e a atividade da amilase salivar mostraram um aumento significativo durante os cenários de teste.
	GC: Treinamento de simulação clássica (MED).	GC: 12			Antes: (5.2 ± 2.7). Imediatamente após: (8.0 ± 6.0). 15 minutos após: (13.2 ± 12.2).	Gerenciamento de tarefas: pré (12.0 ± 3.8) / pós (14.7 ± 4.9). Tomada de decisão: pré: 6.7 ± 2.4) / pós 8.6 ± 2.7). Desempenho Geral: pré (5.6 ± 1.6) / pós: 6.6 ± 1.6).		

2016, Lee et al., Coreia do Sul^(28)^.	GI: Simulação de parto.	GI:12	Investigar se o estado emocional, medido pelos níveis de cortisol salivar de estudantes do último ano de enfermagem, poderia predizer sua aquisição de conhecimento e autoconfiança.	Cortisol Salivar.	Conhecimento: B: 0.15 t: 2.63 (p = 0.17). Auto confiança: B: 0.10 t: 0.31 (p = .7590).	Conhecimento: 2.00 (1.13). Autoconfiança: 6.17 (6.00).	Enfermagem.	Os alunos participantes da simulação de parto obtiveram maior aquisição de conhecimentos e confiança e está associado a níveis mais altos de cortisol.
	GC: Assistir vídeo do processo de parto normal.	GC: 11				Conhecimento: 0.18 (1.08). Autoconfiança: 0.73 (5.31).		

2013, Pottier et al., Bélgica^(29)^.	GI: Consulta de baixo estresse (LS)	GI: 20	Avaliar o impacto do estresse subjetivo e fisiológico na tomada de decisão e nas habilidades de comunicação de estudantes em contexto de consulta ambulatorial.	Cortisol Salivar.	Dia 1: mudança de pré–cenário: 1.75 (1.01). Dia 2: mudança de pré–cenário: 0.10 (0.73).	Comunicação global: linha de base: 64.9% (21.7) / dia do estudo: 69.7% (12.9). Habilidades clínicas: linha de base: 62.8% (15.0) / dia do estudo: 64.8% (10.8).	Medicina.	O estudo mostrou correlações negativas entre raciocínio clínico e estresse. Os estudantes que exibiram maiores níveis de estresse subjetivo e fisiológico obtiveram menos argumentos para diagnósticos diferenciais.
	GC: Consulta de alto estresse (HS)	GC: 21			Dia 1: mudança de pré–cenário: 1.51 (0.99). Dia 2: mudança de pré–cenário: 3.63 (0.71).	Pontuação de comunicação global: linha de base: 55.7% (18.4) / dia do estudo: 68.2% (14.1). Habilidades clínicas: linha de base: 58.0% (11.5) / dia do estudo: 60.9% (10.9).		

GI: Grupo de intervenção; GC: Grupo controle; SIM: Simulação de Alta Fidelidade; SP: paciente padronizados; HFS: Simulação de Alta Fidelidade, LFS: Simulação de Baixa Fidelidade; CS: Cortisol; IQR: Intervalo interquartis; IC: Intervalo de confiança; CHECKLIST: Ferramenta de verificação institucional de avaliação de desempenho; GRS: Escala de classificação global; ANTS: ferramenta de avaliação de habilidades não técnicas de anestesista; FHT: Formulário padronizado de histórico do trauma; NRP: avaliação de megacódigo avançado.

### Risco de Viés dos Estudos

A ferramenta de avaliação crítica utilizada foi a *Collaboration Risk of Bias Tool* (*RoB 2 tool*)^(21)^ a essa ferramenta de avaliação para avaliar o risco de viés dos estudos incluídos, a qual permite avaliar o processo de geração de sequências, ocultação de alocação, ocultação de participantes, pessoal e avaliadores, dados de resultados incompletos e relatórios seletivos dos ensaios clínicos randomizados. Dois pesquisadores avaliaram de forma independente a qualidade de cada estudo, e qualquer discordância seria resolvida pelo terceiro pesquisador.

## RESULTADOS

As buscas realizadas nas oito bases eletrônicas de dados utilizadas nesta revisão sistemática recuperaram 8.514 artigos. Após a remoção dos duplicados, 3147 artigos ficaram disponíveis para triagem. A partir disso, 61 estudos foram selecionados para leitura completa, dos quais 11 preencheram todos os critérios de elegibilidade para esta revisão. O processo de busca e seleção dos estudos está detalhado na Figura 1.

**Figura 1:** Fluxograma do processo de busca na literatura e critérios de seleção dos estudos (adaptado do *Preferred Reporting Items for Systematic Reviews and Meta-Analyses* – PRISMA 2020), Brasília, DF, Brasil, 2024.

Todos os estudos incluídos são ECR e usaram o CS como padrão para mensuração do estresse. Os estudos mencionaram medidas que poderia influenciar na reativação do cortisol. Dentre essas medidas, estavam: progesterona e estrogênio em amostras salivares^(22)^, infecções gerais, doenças do sistema imunológico, doenças endócrinas ou metabólicas, alergias, medicações em uso (exceto anticoncepcionais orais), histórico de doença neurológica ou transtorno psiquiátricos, tabagismo, consumo regular de álcool e drogas, prática de esporte extenuantes, indivíduos que se preparavam para exames seis semanas antes do estudo, bem como gestante, e os que tinham realizado exame nas últimas seis semanas^(9)^, condições médicas envolvendo o eixo hipotalâmico-hipofisário-adrenal, exposição recente a glicocorticoides exógenos, mineralocorticoides, esteroides anabolizantes^(23)^, doenças endócrinas, gravidez, medicamentos como esteroides inalatórios, sistêmicos e betabloqueadores^(24)^.

Além disso, algumas orientações foram dadas aos participantes dos estudos, como se abster de comer, beber líquido contendo cafeína e sucos de frutas, fumar e dormir 4 horas antes da participação do estudo, não beber álcool ou fazer qualquer atividade pesada 24 horas antes de cada sessão experimental^(9)^, não realizar o consumo de alimentos, álcool e/ou nicotina meia hora antes da avaliação e não praticar exercícios 24 horas antes da coleta^(25)^, abster-se de comer e beber por 1 hora antes do período do estudo^(9,10,22)^ e realizar enxague da boca com água 10 minutos antes da coleta da amostra^(22)^.

Os estudos foram publicados no período de 2009 a 2017^(6,9,10,22–29)^, realizados nos Estados Unidos^(23)^, Canadá^(6,10,22,24)^, Singapura^(26)^, Alemanha^(9,27)^, Coreia do Sul^(28)^ e Bélgica^(25,29)^.

Além disso, dez estudos utilizaram algum tipo de simulador^(6,9,10,22-24,26-28)^ e outros dois estudos utilizaram pacientes padronizados^(25,29)^.

Os participantes eram estudantes de enfermagem^(28)^ e de medicina^(9,23,29)^, médicos de Unidade de Terapia Intensiva (UTI)^(27)^, residentes de medicina e de emergência^(10)^, residentes em pediatria^(22)^, residentes neonatais e perinatais^(6)^, residentes em oncologia^(25)^, residentes em anestesiologia^(26)^, e residentes em UTI^(24)^. Nove estudos foram financiados^(9,10,22–27,29)^.

A duração das sessões de simulação variou entre os estudos incluídos, sendo de 10 minutos^(6,27)^, 15 minutos^(9,28)^, 20 minutos^(25)^, ou de 12 a 15 minutos^(26)^.

O desempenho técnico foi medido objetivamente utilizando-se uma lista de verificação relacionada a um cenário^(10,23,27,29)^, diretrizes do Conselho Europeu de Ressuscitação^(9)^, avaliação de megacódigo avançado (NRP)^(22)^, ou utilizando-se o exame de licenciamento de enfermagem coreano^(28)^. Além disso, um estudo avaliou o desempenho subjetivamente, por meio de um questionário autorrelatado^(25)^.

As habilidades não técnicas foram avaliadas por meio de uma ferramenta de avaliação de habilidades não técnicas de anestesista (ANTS)^(6,10,23,26,27)^, escala global de gerenciamento de recursos de crise (Ottawa GRS)^(24)^ e por meio de uma escala likert para avaliação cognitiva^(10)^.

### Síntese dos Dados

Todos os estudos analisados avaliaram o estresse dos participantes utilizando medidas fisiológicas. Outras medidas também foram utilizadas para avaliar o estresse, como medidas psicológicas e autorreferidas. O risco de viés dos estudos foi avaliado como baixo, alto ou pouco claro (Figura 2).

**Figura 2:** Avaliação metodológica de estudos incluídos com base na ferramenta Cochrane^(21)^. Brasília, DF, Brasil, 2024.

Não foi encontrada associação entre o desempenho e estresse em cenários de baixa fidelidade comparados com cenários de alta fidelidade (p = 0,17)^(6)^, nem em cenários de reanimação (p = .098)^(24)^. Entretanto, em outros estudos, os participantes tiveram um desempenho significativamente pior na condição de alto estresse (p < 0,012), indicando que a situação de alto estresse pode ser vista como uma ameaça, levando a prejuízos no desempenho^(10)^. Em consultas ambulatoriais simuladas, os estudantes de medicina obtiveram efeitos deletérios no raciocínio clínico em condição de alto estresse^(29)^.

Nos cenários de ressuscitação neonatal pós-parto com simulação com morte (p = 0,23) ou sobrevivência (p= 0,33), o desempenho foi semelhante nos residentes de medicina de 1° e 2° ano comparado com o desempenho de residentes do 3° e 4° ano^(22)^. Em outro estudo, não houve diferença estatisticamente significativa entre os estudantes de medicina entre o grupo de morte comparado a sobrevida (p = 0,89)^(23)^.

Adicionalmente, em um cenário de emergência de alta fidelidade comparado ao estresse laboratorial, o cortisol aumentou em ambas as condições, porém, nenhuma associação entre o estresse e desempenho durante a simulação foi encontrada (p = 0,631)^(9)^. Já em outro estudo de simulação de emergência, após o treinamento de um dia, os participantes produziram estresse significativo e o desempenho melhorou (p < 0,01)^(27)^.

Em estudantes de enfermagem, níveis mais altos de cortisol foram associados à maior obtenção de conhecimento em um cenário de treinamento e simulação de parto (p < 0,001)^(28)^. O treinamento de habilidades de comunicação tem um efeito na excitação fisiológica. Após treinamento simulado, o nível de cortisol aumentou significativamente em comparação com o grupo controle, melhorando a autoeficácia e as habilidades de comunicação (p = 0,026)^(25)^. Já em outro estudo, o desempenho foi semelhante em habilidades não técnicas entre os papéis ativos *versus* observadores^(26)^.

A avaliação do risco de viés foi realizada por estudo. Em relação aos estudos selecionados, um estudo^(6)^, apresentou alto risco de viés em dois domínios pois havia uma diferença entre o sexo dos participantes (maior participantes do sexo feminino) e os grupos tiveram “pistas” fornecidas pelos facilitadores no cenários, porém as pistas podem ter sido menos obvia em um dos grupos o que pode ter criado uma discrepâncias na compreensão dos participantes. Em outro estudo^(24)^, apresentou baixo risco de viés e risco de viés incerto, a população do estudo foi heterogênea (participantes de vários níveis de formação e especialidades) e a pessoa que supervisionava as sessões de simulação dava feedback aos residentes. Esse fato pode ser sido percebido como fonte de estresse e ter influenciado os resultados. Em outros, demostrou risco de viés incerto, os participantes eram de vários níveis de treinamento^(10)^; contia informações insuficientes sobre o processo de randomização^(25,29)^; houve a exclusão de um participante pois o cortisol estava 10 vezes maior; não foi relatado no estudo nenhum teste de sensibilidade^(26)^; a alocação dos participantes foi por sorteio aleatoriamente^(27)^. Outro fator que produziu alto risco de viés, foi o fato que aproximadamente 29% dos participantes que consentiram com o estudo não completaram as sessões de simulação havendo uma perca de seguimento dos participantes. Não sabemos se todos os eventos de interesse foram adequadamente capturados e pontuados corretamente^(22)^ e a seleção voluntária foi demostrada como risco de viés^(28)^.

## DISCUSSÃO

Esta é uma revisão sistemática sobre as evidências disponíveis acerca do nível de cortisol e desempenho de participantes em simulações realísticas, avaliadas em 11 ensaios clínicos randomizados.

A simulação de alta fidelidade tem se mostrado significativamente estressante, evidenciada pelo aumento dos níveis de cortisol^(9,10,22-27,29)^. Já em outros estudos, os cenários de alta e de baixa fidelidade desencadearam respostas de estresse significativas^(6,24,30,31)^, sugerindo que a simulação de alta fidelidade não seja superior à de baixa fidelidade^(6)^. Adicionalmente, na simulação de média fidelidade o cortisol aumentou significativamente^(28)^.

A respeito de tendências dos estudos ao longo do tempo, pode-se observar que houve um aumento no número de estudos de comparação de alta fidelidade e baixa fidelidade, como de simulação de alto estresse e baixo estresse, o campo emergente tem sido a área da medicina. No que tange os subgrupos, a população do estudo em sua grande maioria era contida por residentes das diversas áreas da medicina e de estudantes de medicina.

Em alguns estudos, não foi encontrada correlação entre marcadores fisiológicos do estresse e o desempenho dos participantes^(6,9,22-24,26)^. Nesse contexto, é fundamental reconhecer que a ausência dessa correlação do estresse e o desempenho em determinados estudos pode ser atribuídas a diversas condições. Por exemplo, a heterogeneidade da amostra, composta por participantes de distintos níveis de formação e especialidades diversas, podendo variar o desempenho devido a diferentes formações. Além disso, a natureza variada dos estresses utilizados nos estudos pode ter desencadeado respostas fisiológicas e psicológica divergentes entre os participantes, resultando em efeitos distintos sobre o desempenho e, consequentemente, impactando de forma significativa os resultados^(24)^.

Além disso, é importante destacar que a não conclusão das sessões de simulação por parte de alguns participantes e o não cegamento dos revisores para natureza do estudo, e para a identificação dos participantes e do cenário são fatores adicionais que podem ter introduzido vieses potenciais na avaliação do desempenho^(21)^. O reduzido número de participantes e representação maioral de integrantes do sexo feminino pode influenciar a validade externa dos achados^(6)^, uma vez que as respostas do estresse e os mecanismos de adaptação pode variar entre os gêneros. Outro ponto a ser colocado, em relação as pistas fornecidas pelos facilitadores aos participantes no cenário de simulação, talvez essas pistas possa ter sido menos óbvias entre os grupos, criando discrepâncias potenciais entre a compreensão dos participantes do estado fisiológico subjacente do paciente^(6)^. Essas questões metodológicas, precisam ser devidamente consideradas ao interpretar os resultados, a fim de garantir uma compreensão mais abrangente e precisa das relações entre estresse e desempenho nos contextos específicos abordados.

Entretanto, outros estudos identificaram que altos níveis de estresse agudo podem prejudicar criticamente a tomada de decisão médica^(10)^ e foram associados a alterações no raciocínio clínico, fazendo que os médicos tivessem menor capacidade de estabelecer diagnósticos^(29)^.

Todavia, em outros estudos, o estresse mostrou-se benéfico para o desempenho dos participantes. Níveis altos de estresse resultaram em melhoria no desempenho clínico e não técnico^(27)^, no conhecimento básico^(28)^ e melhoria na comunicação de más notícias^(25)^.

As respostas ao estresse, determinadas pela percepção de demandas e recursos do indivíduo^(10)^ são consideradas ideais para detectar sinais de alerta e atenuação às respostas. As habilidades de enfrentamento podem ser melhoradas para manter a alostase, enquanto o enfrentamento ineficaz, relacionado a alterações na regulação e responsividade do eixo hipotálamo-hipófise-adrenal (HPA) e liberação do hormônio cortisol, estão associados ao desempenho prejudicado^(32,33)^.

O impacto do estresse agudo no desempenho ainda é discutido^(34)^. Não compreender integralmente o impacto do estresse dos alunos no desempenho do treinamento, envolve o perigo de prejudicar a aprendizagem e a aquisição de habilidades clínicas durante o treinamento, podendo resultar em uma preparação inadequada dos indivíduos para lidar com situações reais^(34)^. Já se tem conhecimento que o desempenho em situações de alta acuidade pode ser melhorado ou prejudicado, dependendo da percepção da demanda e recursos dos indivíduos^(35)^ assumindo que o desempenho aumenta com o nível de estresse até um determinado limite além do qual o desempenho diminui, sugerindo que o estresse coloca a pessoa em ponto de déficit cognitivo^(36)^.

Apesar da importância inerente do ensino por simulação, a carência de experiência e domínio emocional pode desencadear uma reação de estresse, potencialmente impactando o desempenho do aluno^(37)^. A antecipação de situações críticas e a percepção de ser observado induzem a ativação do sistema nervoso autônomo (SNA) e do eixo hipotálamo-hipófise-adrenal (HPA) associado às funções corticais superiores^(38)^. A resposta simpática do sistema autônomo leva ao aumento da pressão arterial, frequência cardíaca, temperatura da pele e metabolismo anaeróbico, enquanto a ativação do (HPA) resulta no aumento da secreção de cortisol no sangue, que é então difundido na saliva durante um período de minutos^(34,38,39)^ o aumento nos níveis de cortisol exerce impacto sobre regiões cerebrais intimamente relacionadas aos processos cognitivos, incluindo a amígdala, o hipocampo e o córtex pré-frontal^(34)^.

Portanto, o treinamento de gerenciamento de estresse tem se mostrado eficiente para a sua redução^(40)^, tendo efeitos positivos não somente nos indicadores do estresse, mas também no desempenho^(41-43)^.

O estresse agudo pode ser um fator de risco para erros de diagnósticos^(29)^ e prejuízo na segurança do paciente^(10)^. Em contrapartida, pode melhorar o desempenho clínico e as habilidades não técnicas^(27)^, pode preparar residentes a lidarem com a morte^(22)^, pode aperfeiçoar as habilidades de suporte avançado de vida^(23)^, a retenção e a consolidação do conhecimento^(26)^, bem como oferecer maiores habilidades clínicas no trabalho de parto^(28)^ e na comunicação verbal^(25)^.

Os efeitos do estresse dependem de vários fatores, sendo eles: sexo, experiência anterior, traços de personalidade, avaliação psicológica, papel atribuído e atribuição da equipe^(10,33)^. Porém, ainda existe dificuldade para saber a origem precisa do estresse^(44)^.

Em relação aos estressores adicionais, a presença de observadores, filmagem, dinâmica de equipe e a percepção de avaliação podem ter influenciado o estresse^(6,26,31)^. Porém, em uma simulação de cirurgia laparoscópica os ruídos não causaram alterações no nível de estresse^(45)^. Em outro estudo^(46)^, distrações como: ligações telefônicas na simulação causaram mudanças nos parâmetros fisiológicos. No estudo de Piquette et al.^(24)^, esses estressores pareceram serem fracos para provocar uma resposta de estresse entre os residentes habituados com um ambiente agitado. Diante disso, os estressores em si podem não levar a prejuízos no desempenho^(10)^. O desempenho pode ser impactado pelo estresse mental, com o acréscimo de elementos ao cenário que levam o sistema cognitivo a correr o risco de sobrecarga. Nesse contexto, em episódios estressantes, a atenção pode ser direcionada exclusivamente para tarefas específicas, resultando na possível negligência de outras informações potencialmente relevantes^(18)^. Sendo assim, é necessário cautela para desenhar os cenários, tendo o cuidado de eliminar o maior número possível de distrações potenciais desses ambientes clínicos^(10)^ tendo conhecimento do mecanismo do estresse e oferecendo recursos para seu gerenciamento^(46)^.

A respeito da exclusão de participantes nos estudos devido a condições que poderiam influenciar os níveis de cortisol, esse fator pode apresentar implicações importantes para a generalização dos resultados e pode introduzir potenciais vieses nas conclusões. Ao restringir a participação com base em fatores relacionados ao cortisol, como condições médicas ou a utilização de medicamentos que afetam a regulação hormonal, há o risco de limitar a representatividade da amostra. Por outro lado, a exclusão seletiva de participantes pode resultar em uma amostra mais homogênea, subestimando ou superestimando os efeitos do cortisol. Dessa maneira, os pesquisadores devem estar atentos e serem cuidadosos a todas as condições que podem modular o cortisol diminuindo o risco de viés de confusão.

Quanto às limitações dos estudos, é importante ressaltar o viés de cegamento dos participantes e dos avaliadores e a falta de um instrumento de avaliação do desempenho validado e adequado para os cenários, impossibilitando a generalização dos resultados e o pequeno tamanho amostra pois não fornece poder estatístico necessário. Alguns estudos apresentaram, em sua amostra, participantes de diferentes níveis de experiência, formação, especialidade e a população amostral foi escolhida por conveniência ou incluída de forma voluntária o que pode impactar negativamente no nível das evidências dos estudos. Além do mais, grande parte dos estudos não abordou a experiência previa dos participantes com a simulação.

Em relação, às limitações inerentes a resta revisão, destaca-se a ausência da realização de uma metanálise, devido a heterogeneidade dos estudos incluídos em relação a: método, desenho amostral e análise estatística. Houve também desafios relacionados ao acesso a dados em alguns estudos, e, lamentavelmente, ao tentar entrar em contato com o autor responsável do artigo, não obtivemos sucesso. Essa dificuldade pode ter resultado na possível perda de informações relevantes que poderiam contribuir para a inclusão e compreensão desta revisão.

A revisão apresentada permitiu uma ampliação dos conhecimentos no que tange a associação entre os níveis de cortisol e o desempenho em simulação clínica, destacando a complexidade das interações entre o sistema endócrino e o desempenho na prática em ambientes simulados.

Os resultados podem ser essenciais para professores e profissionais que trabalham com simulação clínica, esses devem levar em consideração o nível de estresse do participante e as condições que modulam o estresse, visto que a regulação do cortisol pode desempenhar um papel crucial na adaptação e no desempenho do participante. Além do mais, precisam ter cautela ao desenhar um cenário clínico, conhecer os fatores que afetam o desempenho pode contribuir para a melhoria da prática clínica e aprimoramento da qualidade da assistência à saúde. Além disso, deve-se levar em consideração o gerenciamento do estresse para que a simulação não seja um trauma, mas uma forma de aprendizado ao aluno.

## CONCLUSÃO

Até onde se sabe, esta é a primeira revisão sistemática sobre o impacto do cortisol em relação ao desempenho em simulações. À medida que os níveis de cortisol se alteram, o desempenho dos participantes muda, de forma prejudicial ou benéfica. Porém, em outros estudos não houve correlação entre estresse e desempenho que pode não ter ocorrido por questões metodológicas. É notório a carência de evidências científicas robustas no cenário em questão, ressaltando a necessidade premente de pesquisas mais criteriosas e bem delineadas. A respeito da lacuna de pesquisa, não se sabe até que ponto o estresse pode ser benéfico ou prejudicial para o desempenho e se essa variação é mutável de acordo com o nível de dificuldade do cenário ou estressores do cenário de simulação.

Sobre futuros estudos, sugere-se a realização de ensaios clínicos randomizados, bem delineados para que diminuam os riscos de vieses e que abranjam uma ampla gama de fidelidades, englobando desde ensaios de baixa até alta fidelidade que avaliem a causalidade entre a exposição e desfecho, aumentando significativamente o tamanho amostral e tendo populações distintas em sua amostra.
